# Advances in Corneal Tissue Engineering: Comparative Performance of Bioengineered Grafts in Animal Models

**DOI:** 10.3390/medicina62010080

**Published:** 2025-12-30

**Authors:** Eduardo Anitua, Mar Zalduendo, Mohammad H. Alkhraisat

**Affiliations:** 1 Regenerative Medicine Department, BTI Biotechnology Institute, 01007 Vitoria, Spain; marimar.zalduendo@bti-implant.es; 2 University Institute for Regenerative Medicine and Oral Implantology-UIRMI (UPV/EHU-Fundación Eduardo Anitua), 01007 Vitoria, Spain; 3 Department of Oral and Maxillofacial Surgery, Oral Medicine and Periodontology Faculty of Dentistry, University of Jordan, 11942 Amman, Jordan

**Keywords:** cornea transplantation, bioengineered cornea, corneal graft, tissue regeneration, tissue engineering

## Abstract

*Background and Objectives*: Corneal opacity is the fifth global cause of blindness and moderate-to-severe visual impairment due to scar tissue formation. The purpose of this study is to provide an integrated overview of the current state of corneal engineering strategies focused on the comparison with healthy corneas. It aims to identify engineering strategies that would result in functional corneas, providing real alternatives to donor corneal transplants. *Materials and Methods*: systematic review was conducted according to the Preferred Reporting Items for Systematic Reviews and Meta-Analyses (PRISMA) and according to the protocol with the ID: CRD420250654641 at the PROSPERO database. The focus question, prompted by considering the shortage of human corneal grafts, was: what is the performance of bioengineered corneal grafts in experimental animal models when compared with healthy eyes in the restoration of corneal anatomy and function? *Results*: Incorporating human corneal epithelial cells w/ or w/o human corneal stromal stem cells into a gelatin methacrylate and polyethylene glycol diacrylate matrix emerges as the leading option for epithelial layer regeneration. Human and bovine decellularized corneas, porcine corneal ECM in Gelatin methacrylate, dual layered collagen vitrigel and tissue-engineered human anterior hemi-corneas have shown promise for simultaneous regeneration of the corneal stromal and epithelial layers. Corneal stromal tissue regeneration could be positively impacted by transplantation with grafts derived from aligned self-lifting analogous tissue equivalents and collagen-based hydrogels. Finally, scaffolds of silk fibroin and human purified type I collagen represent promising approaches for corneal endothelial regeneration, though their effectiveness is contingent upon integration with endothelial cells. *Conclusions*: Collectively, these findings contribute to the growing body of evidence supporting the potential of tissue-engineered corneal substitutes as viable therapeutic options for corneal blindness and vision impairment. Assessing the optical and functional properties of the regenerated cornea should be a cornerstone in all studies aiming to evaluate their clinical effectiveness.

## 1. Introduction

Corneal opacity is the fifth global cause of blindness (contribution of 3.46%) and moderate-to-severe visual impairment (MSVI) (1.29%) [1]. It has been estimated that 11.7 million people worldwide have MSVI or blind (in at least one eye) due to corneal opacity [2]. Vision loss or impairment arises from inadequate resolution of a corneal lesion, leading to scar tissue formation and light scattering at the cornea [3,4]. Traumatic, chemical, infectious and surgical injuries, along with corneal disorders and diseases, can lead to corneal opacity. Visual impairment severely affects the quality of life of the population and represents an enormous economic burden [5]. The global annual loss in productivity due to blindness has been estimated at $43.6 billion (95% uncertainty interval: $34.4–54.5 billion), while MSVI contributed $367.1 billion ($287.7–464.2 billion). This economic burden is largely preventable through the development of effective prevention programs and treatment strategies, which could enhance global economic productivity and contribute to poverty reduction [5].

Depending on the severity of corneal lesions various treatment options are available, including pharmaceutical drugs, plasma rich in growth factors eye drops and corneal transplantation. Although corneal transplantation is the most commonly performed transplant procedure worldwide, it is hindered by a global shortage of donor tissue, the risk of immune rejection and its limited applicability in certain patient populations [6]. Gain et al. have described the significant shortage in corneal transplant worldwide, estimating the availability of only one cornea for every 70 patients in need of transplantation [7]. Moreover, the rejection of the transplanted cornea occurs in 10% of cases that could increase in high-risk patients [8,9]. These limitations highlight the need to find alternative solutions to the shortage of corneal donations. In this context, tissue engineering has emerged as a highly promising field, offering innovative solutions for the repair and replacement of injured tissues and organs [10,11].

An effective bioengineered corneal graft should be capable of restoring the cornea’s essential functions, including light transmission and refraction, as well as protection against mechanical injury, ultraviolet radiation and microbial infections [10]. Structurally, the human cornea is composed of three cell layers, including the corneal epithelium (outer layer), the stroma and the endothelium (inner layer), which are separated by two acellular interfaces [12]. Bowman’s layer is a condensed, acellular collagenous layer measuring approximately 8–12 μm in thickness, located between the stratified epithelium and the stromal layer, and plays a role in regulating molecular transfer [12]. Descemet’s membrane, on the other hand, is the basal lamina of the corneal endothelium, acting as a boundary between the endothelium and the stroma [13]. Thus, each cellular layer of the cornea exhibits a distinct cellular composition and presents complex challenges for tissue engineering [11].

Scaffold-based and scaffold-free strategies represent two principal bioengineering approaches for tissue reconstruction, both of which have experienced significant advancements over the past decade [14]. Cellular scaffold-free approaches enable the delivery of high cell densities to the target site and would be particularly suitable for reconstructing the corneal epithelium and endothelium, as these layers are characterized by minimal extracellular matrix complexity [15,16]. Cells in this approach produce a 3D matrix that remodels and guides tissue formation [14,17]. In addition, cellular scaffold-free constructs might reduce post-transplantation cytotoxicity and potentially obviate the need for suturing owing to the intrinsic adhesive properties of the cell-derived matrix and the absence of exogenous biomaterials [18,19]. On the other hand, scaffold-based approaches offer advantages in scalability and manufacturability and could be particularly suitable for full-thickness corneal reconstruction encompassing stromal tissue regeneration [20,21]. These strategies might combine biomaterials, cells and signaling factors in a functional matrix that supports tissue regeneration. The design, mechanical properties, surface chemical characteristics, adhesive behavior and biological functionalities of the scaffolds are governed by the intrinsic physicochemical properties of the constituent materials, the manufacturing processes and the possible strategic integration of cellular components, bioactive molecules and cross-linking agents. Scaffold-based constructs might influence cell antigen expression, adhesion, proliferation and matrix formation and could be designed to promote specific bio-integration [22]. The ultimate goal is to provide an alternative that meets not only the optical requirements of the cornea, such as transparency to visible wavelengths and the capacity to establish a near-perfect optical interface for precise refraction of light onto the retina, but also its protective role [23]. Corneal tissue substitutes should have adequate viscoelastic properties to absorb mechanical stress and maintain structural integrity [11].

To date, a variety of biomaterials have been explored as biological substitutes for the corneal tissue, notably including collagen-based membranes, decellularized corneal stroma, chitosan, polyglycolic acid (PGA) and fibrin-derived scaffolds, among others [24,25,26,27], due to their favorable structural and biochemical properties [23]. Despite notable advancements, current scaffold designs remain insufficient to simultaneously replicate the biochemical composition, mechanical integrity, structural organization, optical properties and hierarchical architecture of the native corneal stroma within a single construct [24,28].

Numerous reviews have been published evaluating the current state of corneal tissue engineering with the vision to provide evidence on effective bioengineering strategies for corneal tissue substitutes. These reviews focus on specific aspects of corneal tissue engineering, such as individual corneal layers [29], distinct scaffold types or biomaterials [18,19,30,31,32], particular cell or regenerative strategies [33,34,35], specific fabrication technologies [20,21], or targeted disease conditions [36]. Although some reviews provide a more comprehensive overview of different approaches for corneal regeneration, from conventional transplantation to advanced tissue engineering, they are not systematic reviews and lack bias assessment of the included studies [12,24,37]. Furthermore, the current body of knowledge can be strengthened by providing an evaluation based on the outcomes derived from in vivo comparisons with native healthy corneas. To bridge this gap and generate robust evidence that facilitates clinical translation, this review presents the recent advances in regenerative strategies for corneal bioengineering, focusing on comparative analyses against native healthy corneal tissue to assess the efficacy of these strategies in restoring the corneal physiological function and structure. Evidence, derived from studies previously subjected to systematic bias assessment, has been categorized according to the specific corneal layers targeted for regeneration, examining the interplay between scaffold type, cellular components and in vivo implantation outcomes. Finally, this review will provide a comprehensive resource for researchers in the field of tissue engineering and ophthalmology offering an integrated overview of the current state of corneal regeneration strategies focused on the comparison with healthy corneas. It aims at next-generation engineering strategies that would result in functional cornea providing a real alternative to donor corneal transplants.

## 2. Materials and Methods

This systematic review was conducted according to the Preferred Reporting Items for Systematic Reviews and Meta-Analyses (PRISMA). The protocol has been registered in the International prospective register of systematic reviews PROSPERO database with registration ID: CRD420250654641, and no protocol amendments were implemented following registration.

### 2.1. Focus Question

Considering the shortage of corneal grafts from cadaveric donors for the treatment of corneal blindness, the aim of this review is to gather the performance of bioengineered corneal grafts in experimental animal models when compared with healthy eyes in the restoration of corneal anatomy and function.

### 2.2. PICO Strategy

Research strategy was framed following the PICO structure:

- Population: Any animal model of corneal damage susceptible to keratoplasty, without species limitation;
- Intervention: Transplantation of corneal grafts to assess the efficacy of these structures in terms of restoring corneal function;
- Comparison: Studies where lateral or contralateral controls such as the healthy eye were considered;
- Outcomes: Parameters for assessing graft efficacy in terms of recovery of corneal functionality and/or biocompatibility such as corneal transparency or thickness measurements among others.

### 2.3. Search Strategy

For this systematic review, the literature search was performed in PubMed, Scopus and Web of Science databases on 19 February 2025, using the following search strategies: ((bioengineered) OR (artificial) OR (biomimetic)) AND (cornea) AND ((cornea restoration) OR (cornea regeneration) OR (cornea transplantation)) AND (human) AND (in vivo) for PubMed, (TITLE-ABS-KEY (bioengineered) OR TITLE-ABS-KEY (biomimetic) OR TITLE-ABS-KEY (artificial) AND TITLE-ABS-KEY (cornea) AND TITLE-ABS-KEY (in AND vivo) AND TITLE-ABS-KEY (human)) AND PUBYEAR > 2014 AND PUBYEAR < 2026 AND (LIMIT-TO (DOCTYPE, “ar”) OR LIMIT-TO (DOCTYPE, “re”) OR LIMIT-TO (DOCTYPE, “le”)) for Scopus and ((((ALL = (bioengineered)) OR ALL = (biomimetic)) AND ALL = (cornea)) AND ALL = (in vivo)) AND ALL = (human) for Web of Science. Results from the search were restricted to studies published in the past 10 years.

Considering the selection criteria, studies in which graft efficacy assessment was tested in any animal model of corneal damage susceptible to keratoplasty, without species limitation were included. The following exclusion criteria were applied: (a) articles not published in English or Spanish, (b) reviews, book chapters…, (c), corneal grafts in droplet or liquid form, (d) out of scope, (e) duplicates, (f) sample size less than 5 or not described, (g) no comparison with healthy eyes, (h) full text was not available.

### 2.4. Data Extraction

An initial screening was performed based on the title and abstract to determine the suitability of the articles. Studies that passed this first evaluation were retrieved for full-text review. For data extraction, an evidence table was created with Microsoft Excel in which the following data were included: (a) article information (authors and year of publication), (b) animal species (sample size), (c) graft tested, (d) scaffold composition, (e) Toxicologic/biocompatibility assays, (f) cell phenotype, when appropriate, (g) previous in vitro assays for ensuring cell viability, when appropriate, (h) scaffold suturability, (i) bioprinting approaches, (j) corneal layer to be restored, (k) keratoplasty description, (l) comparison groups, (m) maximum follow-up duration, (n) parameters for graft efficacy assessment and (o) final outcomes compared with healthy eye.

### 2.5. Risk of Bias Assessment

Studies were evaluated with SYRCLEs RoB tool, an adapted version of the Cochrane risk-of-bias tool [38]. Ten items: (1) allocation sequence generation, (2) baseline characteristics, (3) allocation concealment, (4) random housing, (5) blinding, (6) random outcome assessment, (7) blinding, (8) incomplete outcome data, (9) selective outcome reporting and (10) other source of bias), categorized under five domains (Selection bias (1, 2, 3), Performance bias (4, 5), Detection bias (6, 7), Attrition bias (8), Reporting bias (9) and Other (10)), were assessed with “yes”, “no” or “unclear” if insufficient details have been reported and a judgment of low, high or unclear risk of bias was assigned to these three options, respectively. Two reviewers individually evaluated the risk of bias, using the coincident standard and any disagreement was addressed by sufficient discussion. When consensus was not reached, a third person was consulted.

## 3. Results

**Figure 1:**
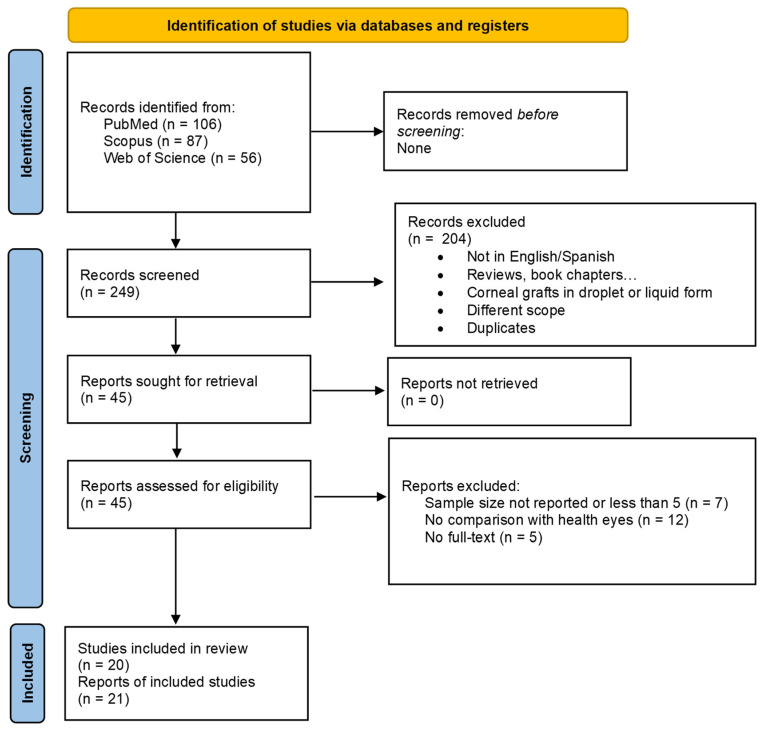
Flow chart diagram showing the screening and selection of the studies in this systematic review.

The search strategy yielded a total of 249 articles from the three databases. After an initial screening, 204 references were removed: 64 due to duplication and the rest either because they were review articles, their objectives did not align with the aim of this systematic review or because they involved droplets or liquid grafts. After exhaustive screening, 21 studies (20 articles) [39,40,41,42,43,44,45,46,47,48,49,50,51,52,53,54,55,56,57,58] were finally included for analysis in this review (Figure 1). The research excluded were those where sample size was not described or was less than five animals (7), those with no full text available (5) and those where comparison with healthy eyes was not established (12).

Table 1 summarizes selected preclinical studies that investigated bioengineered scaffolds for corneal tissue regeneration in animal models, in which the healthy eye was included as a control. The table classified the studies according to the corneal layer targeted for regeneration and emphasizes scaffold composition, bioprinting approaches, toxicologic or biocompatibility assays and graft suturability. Cell phenotypes and assays evaluating cell viability or functionality are shown where appropriate. All studies utilized rabbit models, except for those conducted by Xu B et al. in 2017 [58] and Syed-Picard FN et al. in 2018 [47], which tested a tissue-engineered anterior hemi-cornea in beagle dogs and bioengineered tissues sheets of corneal stromal stem cells (CSSC) differentiated to keratocytes in C57BL/6 mice, respectively.

Studies were classified according to the specific corneal layer targeted for regeneration, in order to strengthen the evidence on graft efficacy depending on its clinical applicability.

### 3.1. Corneal Epithelial Layer

Three studies evaluating corneal epithelium regeneration in rabbits following artificial graft transplantation and meeting the inclusion criteria were included in this review. Limbal stem cell deficiency models were established prior to transplantation surgery in all studies. Thus, denuded corneal surfaces and limbal niche removal were achieved by different approaches such as keratectomy combined with the application of 99.9% alcohol [50], ethanol treatment followed by epithelial layer debridement [39], or exposure of the corneal and limbal regions to 0.5 N sodium hydroxide [46]. The conjunctival epithelial layer covering the cornea was removed and a peritomy was performed in cases involving a prolonged interval (8 weeks) between the limbal stem cell deficiency establishment and graft transplantation surgery [50] (Table 2).

Regarding the scaffold composition, GelMA-PEGDA (gelatin methacrylate and polyethylene glycol diacrylate) [39] and PLGA (poly(lactic-co-glycolic) acid) [46] were the biomaterials used, since a scaffold-free cell sheet of human oral mucosal and limbal epithelial cells was applied in the other study [50]. The effectiveness of both GelMA-PEGDA and PLGA materials was examined as well as their combination with human corneal cells. In this sense, GelMA-PEGDA hydrogel with cells embedded (human corneal epithelial cells (CECs) or both corneal epithelial and stromal stem cells (CSSCs)) was used as bioink for fabricating two different 3D bioprinted models (Table 1). On the other hand, human conjunctival epithelial cells (hCECs) were seeded on PLGA membranes. Previous in vitro assays for ensuring cell viability were performed in all three studies (Table 1). Concerning the postoperative suture placement, associated with potential adverse outcomes such as localized inflammation, they were used to attach GelMA-PEGDA and PLGA grafts to the deepithelialized rabbit cornea. In contrast, tissue sheets of cultivated oral mucosal cells were transplanted without suturing, and contact lenses were used to promote graft adhesion.

The length of the observation period from transplantation surgery differed among the three studies. Thus, the efficacy of biomaterial-free cell sheets, PLGA membranes and GelMA-PEGDA biomimetic limbal implants were assessed for up to one, two and four weeks after surgery, respectively.

A range of outcomes was obtained depending on the composition of the scaffolds used for corneal regeneration. A well-adhered graft displaying multiple layers of oral mucosal epithelial cells with no visible goblet cells (typically in conjunctive) was observed for biomaterial-free cell sheets. Grafted cells expressed key markers associated with stem cell-like proliferative potential. When PLGA membranes were applied, they supported re-epithelialization with transplanted corneal epithelial cells although a partially graft degradation was reported. Regarding GelMA-PEGDA limbal implants, histological analysis revealed regularly arranged epithelial cells, with neither neovascularization nor fibroblast-like cells observed, similarly to the control. Furthermore, no goblet cells were detected.

Future perspectives: It would be advisable to evaluate additional graft materials and to perform a more comprehensive characterization of transplanted grafts by assessing transparency and other key parameters relevant to tissue regeneration, such as postoperative inflammation and fibrotic processes, as well as by extending the duration of the observational follow-up.

### 3.2. Corneal Epithelial and Stromal Layers

Based on the scaffold’s physical properties, the ten studies evaluating corneal epithelial and stromal restoration were divided into two groups: those testing injectable hydrogels [40,41] and those in which suturable grafts were analyzed (Table 1). In the first group, cornea wound was filled or covered with the hydrogel graft, eliminating the need for sutures and avoiding the inflammatory response often associated with them. The only precaution required was to ensure the gel remained at the transplantation site. For this purpose, contact lenses were usually placed and the eyelids were stitched [40].

Concerning the scaffold composition, decellularized extracellular matrix obtained from porcine or bovine corneal tissue were mainly used (DCMH [40], CECM-GelMA [41], DPC [42], 3D-BDCS [44], DLPCS [55] and aPCS [58]), followed by gelatin composites (dual-layered collagen vitrigel consisted of biomimetic synthetic Bowman’s membrane (sBM) and a synthetic stromal layer (sSL), containing tissue-derived extracellular matrix (ECM) microparticles [57], rhEGF/TSA-loaded bilayer hydrogel [54]) and nanostructured fibrin-agarose-based material (TEAHC [43], OAC and HAC [56]) (Table 3).

It is noteworthy to mention that 3D printing systems were used to fabricate composite grafts in 30% of cases. Thus, cornea substitutes have been engineered using decellularized corneal tissue (CECM-GelMA and 3D-BDCS) and a collagen-alginate mixture solution (rhEGF/TSA-loaded bilayer hydrogel) as natural bioinks (Table 1).

In addition, more than half of the grafts included stromal fibroblasts or a combination of both corneal epithelial and stromal phenotypes, regardless of the composition and physical properties of the scaffold (Table 1). In two out of these six studies, human primary stromal cells were included into the scaffold, and cell viability for CECM-GelMA [41] or functionality for DPC [42] were in vitro confirmed before graft transplantation. Furthermore, human turbinate–derived mesenchymal stem cells (hTMSCs) differentiated into keratocytes were incorporated into 3D-BDCS scaffolds [44]; however, assays confirming stromal differentiation were not reported. In the remaining half of cases, corneal epithelial cells along with the stromal phenotype were incorporated into grafts. Thus, TEAHC constructs were enriched with primary cell cultures of epithelial and stromal cells whose viability and cell function were verified [43]. Similarly, another two different compositions of cell-enriched fibrin-agarose scaffolds were evaluated: stromal cells embedded within the graft matrix and a stratified epithelial top layer composed of either human corneal epithelial cells (OAC, orthotypical bioartificial corneas) or human Wharton’s Jelly mesenchymal stem cells (HWJSC) (HAC, heterotypical bioartificial corneas) [56]. However, no assays assessing cell viability or functionality within the scaffold were reported. In order to analyze the impact of scaffold cellularization, the inclusion of nontransfected human corneal stromal (ntHCS) and epithelial (ntHCEP) cells in TE-aHC constructs was evaluated by comparing with acellular porcine corneal stromata scaffolds [58]. Furthermore, the specific cellular activity associated with each corneal cell phenotype was confirmed prior to graft transplantation.

Corneal keratectomy was performed mainly by trephination followed by manual dissection to remove the epithelium and underlying stromal layer. In only one case, the graft was inserted into a three-quarter circle incision beneath a surgically lifted flap without corneal tissue resection (3D-BDCS). With respect to the stromal depth of the keratectomy, anterior lamellar tissue removal was performed in 7 out of the 10 studies, whereas the remaining three involved deeper resections (>200 μm) [42,44,57]. All bioengineered grafts were transplanted into rabbit injured corneas, with the exception of TE-aHC, which was subjected to in vivo evaluation in a canine beagle model. Sixty percent of the studies included follow-up periods of at least three months, with a full one-year postoperative assessment performed in some instances (TEAHC, DLPCS, OAC, HAC and TE-aHC)

In vivo assays to evaluate scaffold biocompatibility prior to transplantation were exclusively conducted for the DCMH graft, in which the absence of erythema and edema was confirmed by histological analysis of skin samples collected 72 h after subcutaneous injection. Nevertheless, TEAHC graft potential toxicity was assessed at 12 months post implantation. Thus, no local migration to surrounding tissues nor any sign of systemic cell distribution, toxic effects, local infection, granuloma, tumorigenesis or systemic side effects during the follow-up period, as well as a complete absence of tumors or predisposition to them, were reported for TEAHC scaffolds. Similar assessments were conducted for CECM-GelMA and 3D-BDCS grafts [41,44].

Keratoplasty success, in relation to graft composition, was commonly assessed based on the presence or absence of inflammatory and fibrotic responses, corneal transparency and thickness and, in selected cases, the restoration of native corneal architecture and extracellular matrix composition. In this sense, no sign of inflammatory processes was described for DCMH, CECM-GelMA and engineered dual-layered collagen vitrigel. Post-surgical corneal edema, initially present following the implantation of other analyzed scaffolds, progressively resolved over time, reflecting an acceptable host tissue response and integration. However, marked corneal edema and neovascularization were observed throughout the monitoring period for aPCS grafts. On the other hand, corneal haze was often quantitatively measured using image analysis software, whereas in other cases, transparency was qualitatively evaluated using a slit-lamp biomicroscope based on the visibility of intraocular structures such as the iris. In this sense, adequate graft adherence and sealing to the host corneal bed, along with sustained optical transparency were reported for rhEGF/TSA bilayer hydrogel grafts. Similarly, TE-aHC, DCMH, CECM-GelMA, TEAHC, DLPCS, dual-layered collagen vitrigel, OAC and HAC scaffolds grafts exhibited a gradual increase in corneal transparency becoming nearly indistinguishable from that of normal control eyes at the end of the study time. However, transparency did not return to the cornea when aPCS and decellularized or recellularized DPC grafts were transplanted. In the last two cases, as well as in corneal epithelial and stromal layers after Gel/Alg-CDH hydrogel scaffold transplantation, an extensive staining for the alpha-smooth muscle actin (α-SMA) myofibroblast marker associated with fibrotic processes was described [42,54]. Conversely, no histopathological evidence of fibrosis was induced by DCMH, CECM-GelMA, rhEGF/TSA, dual-layered collagen vitrigel, aPCS and TE-aHC artificial corneas. Finally, appearance of fibrotic processes has not been evaluated following transplantation of TEAHC, 3D-BDCS, DLPCS, OAC and HAC grafts.

Thickness of the stroma and epithelium were commonly calculated from cross-sectional images of cornea tissue obtained by anterior segment optical coherence tomography (AS-OCT) using. No significative differences with respect to the control healthy eye were reported for most of the scaffolds considering this parameter, since a progressive reduction in overall thickness of corneal layer was observed from the early postoperative period onwards (DCMH, CECM-GelMA, rhEGF/TSA, DLPCS, dual-layered collagen vitrigel and TE-aHC and aPCS grafts). Cornea thickness did not return to normal values when 3D-BDCS and Gel/Alg-CDH hydrogel were grafted.

The graft suitability was more accurately analyzed by histologic examination in some engineered scaffolds. Thus, a complete multilayered epithelial covering and a regularly arranged stroma with sparsely distributed stromal cells resembling normal corneal tissue were described for DPC, rhEGF/TSA and TE-aHC. Histological examination resembling control structures were also observed in corneas transplanted with DCMH, TEAHC aPCS and dual layered collagen vitrigel. Similar corneal structure but a dense stroma with abundant cells was exhibited after OAC and HAC graft transplantation. In contrast, epithelial and stromal cell density lower than in normal rabbit cornea were found for DLPCS artificial constructs. Regarding ECM composition, the expression of epithelial and stromal proteins (cytokeratin 3 (CK3), cytokeratin 12 (CK12), Col I, lumican (LUM), keratan sulfate (KER), decorin or/and the distribution pattern of collagen fibrils, were examined in DCMH, CECM-GelMA, rhEGF/TSA, de- and re-cellularized DPC, TE-aHC, aPCS, OAC, HAC and dual-layered collagen vitrigel grafts, confirming the effective corneal tissue regeneration.

Future perspectives: Substantial efforts have been devoted to the development and characterization of artificial grafts for the regeneration of both corneal epithelial and stromal layers. However, the optical properties of the transplanted corneas have not been extensively evaluated. In addition, the potential benefits of incorporating cells into these grafts warrant further investigation.

### 3.3. Corneal Stromal Layer

Six studies, encompassed within five scientific publications, have been reviewed to evaluate the potential of diverse biomaterials and tissue engineering strategies for corneal stromal regeneration. One only study used C57BL/6 mice as experimental animal model [47], while all other investigations selected New Zealand white rabbits for in vivo evaluation (Table 1).

Scaffold materials varied widely, including decellularized porcine corneas, collagen-based hydrogels, reduced graphene oxide-reinforced titania-based composites and corneal analogous tissue based on stromal ECM (A-SLATEs, R-SLATEs). Moreover, implants consisting of human corneal stromal stem cells (CSSC) tissue sheets without any material support [47] have also been evaluated. The efficacy of recellularization process in DPC-HHP implants, obtained after corneal high hydrostatic pressurization followed by DNase I treatment, was assessed in two studies conducted by Hashimoto Y et al. in 2016 [52]. Moving forward with cellularized implants, bioengineered human corneas consisting of ECM synthesized and deposited by isolated human stromal cells were evaluated. More precisely, the effect of distinct implant tissue architectures on corneal stromal layer regeneration was evaluated, specifically aligned (A-SLATEs) versus randomly oriented (R-SLATEs) structures [49]. Determination of cell viability and detection of stromal ECM markers as sign of cell functionality have been performed prior to transplantation surgery of both cell sheet and SLATEs grafts (Table 1). Regarding cell-free scaffolds, collagen-based scaffolds consisted of a central transparent core and an embedded peripheral skirt of adjustable thickness, degree of crosslinking and collagen content (MBPC thin, MBPC thick, HBPC, MHBPC thick) [53] and composites fabricated from a defined mixture of graphene oxide nanoparticles and liquid crystalline graphene oxide with titanium dioxide powder (1rGO/TiO_2_ and 1rLCGO/TiO_2_) [45] have been assessed.

Most of the analyzed scaffolds were suturable, except for cell sheet grafts and collagen-based composites. However, the application of sutures was only necessary to close the incision created for the implantation of 1rGO/TiO_2_ and 1rLCGO/TiO_2_ composites, which were inserted into stromal pockets generated at 75% depth of the corneal stroma using a femtosecond laser. The surgical strategy most commonly used for keratoplasty was the generation of an intrastromal pocket. In this sense, cell sheet implants were grafted using forceps into pockets created using a 27 G needle and without removing any stromal tissue. In contrast, A- and R-SLATEs, DPC-HHP transplanted by interlamellar keratoplasty and collagen-based hydrogel composites (MBPC thin, MBPC thick, HBPC and MHBPC thick) were implanted into stromal pockets which were surgically created either with crescent knives or, in the latter case, via femtosecond laser photo disruption, at a depth corresponding to ≤50% of the total corneal thickness. Stromal tissue was excised using manual microsurgical dissection techniques prior to implantation. Alternatively, a corneal flap was generated at a depth of 200 μm using microkeratome-assisted anterior lamellar keratoplasty in order to assess stromal regeneration following DPC-HHP graft transplantation (Table 4). Regarding the postoperative evaluation period, half of the studies [49,52,53] conducted follow-up evaluations lasting more than two months post-grafting. Observation periods exceeding three months were reported for collagen-based composites, both aligned and randomly oriented SLATEs, as well as decellularized porcine corneas (DPCs) implanted via anterior lamellar keratoplasty.

Biocompatibility and systemic toxicity assessments were exclusively documented for 1rGO/TiO_2_ and 1rLCGO/TiO_2_ scaffolds (Table 1). Postoperative monitoring revealed no evidence of behavioral abnormalities, including appetite suppression, stress-related behaviors, discomfort, or significant fluctuations in body weight, thus supporting the in vivo tolerability of these materials.

Corneas transplanted with acellular DPC-HHP, tissue cell sheets and aligned or randomly oriented SLATEs showed a marked improvement in transparency, which remained stable throughout the follow-up period, whereas the post-operative corneal haze associated with collagen-derived scaffolds (MBPC thin, MBPC thick, HBPC and MHBPC thick) progressively diminished over time.

No inflammatory response was observed following the transplantation of 1rGO/TiO_2_, 1rLCGO/TiO_2_, A- and R-SLATEs and DPC-HHP grafts. However, outcomes related to the fibrotic response exhibited greater variability. Absence of fibrosis markers was described for 1rGO/TiO_2_, 1rLCGO/TiO_2_ grafts. Similarly, no signs of fibrotic processes were observed after A-SLATE transplantation. In contrast, collagen-based hydrogels were highly populated by α-SMA myofibroblasts and fibrosis signs were also found after R-SLATE graft transplantation. More detailed assessments were conducted in certain cases, where no structural differences with respect to normal corneas were found for 1rGO/TiO_2_, 1rLCGO/TiO_2_, cell tissue sheets, composite collagen-based hydrogels (MBPC thin, MBPC thick, HBPC, and MHBPC thick grafts) and DPC-HHP when performing the histological analysis at six months after the anterior lamellar keratoplasty. Regarding ECM components, the preservation of KERA expression in transplanted cell tissue sheets was described. Moreover, vimentin-positive cell levels comparable to those observed in the control healthy tissue were reported for both S-LATE grafts throughout the study period. Light scattering was exclusively assessed in transplanted corneas with cellular tissue sheets, yielding values similar to those observed in non-operated native control corneas.

Future perspectives: Despite the development of a large number of scaffolds, the evaluation of the transplanted cornea has often been insufficient. In particular, parameters such as corneal thickness and optical properties, including light scattering, have frequently not been assessed. Moreover, comprehensive analyses of extracellular matrix composition, confirming appropriate collagen fiber organization and proteoglycan composition required to ensure the characteristic transparency of corneal tissue, are often lacking.

### 3.4. Corneal Endothelial Layer

Two studies carried out by Vázquez et al. (2016, 2017) evaluated two distinct scaffold compositions (human purified type I collagen membrane (HPCM) and silk fibroin (SF)) for bioengineered endothelial grafts in New Zealand white rabbit models [48,51]. The impact of incorporating corneal endothelial cells (CENCs) on the regenerative properties of these suturable scaffolds was evaluated after conducting cell characterization analysis. Moreover, in vitro determinations for ensuring cell functionality were performed prior to SF graft transplantation. In this context, the presence of endothelial markers was confirmed through positive immunostaining, along with the identification of their typical polygonal cellular morphology (Table 1).

Corneal endothelium was removed from the anterior chamber with a 30-gauge needle after incising the limit of the corneoscleral tissue with a slit knife in Descemet’s membrane endothelial keratoplasty (DMEK). Trephined grafts were injected with a small volume of a viscoelastic agent, unfolded into the anterior chamber and fixed to the posterior stroma stripped of Descemet’s membrane. Then, the corneoscleral wound was closed with nylon sutures. At six weeks post-surgery, corneal thickness was assessed using AS-OCT and histological analysis and transparency evaluation were performed.

Eyes transplanted with SF acellular grafts exhibited persistent corneal edema and failed to recover transparency, as can be seen in Table 5. These scaffolds induced mild fibrosis within the corneal stroma and led to an increase in corneal thickness, indicative of impaired corneal endothelial functionality. In contrast, CENCs incorporation into SF grafts resulted in a fully integrated component displaying a similar corneal thickness and endothelial cell count when compared with its healthy contralateral cornea. Moreover, cellularized SF artificial scaffolds restored the corneal transparency and endothelial cell functionality at 6 weeks of follow-up without a significant inflammatory reaction. Similarly, HPCM grafts seeded with rabbit CENCs behaved as a fully integrated component in the corneal tissue. Transplanted corneas maintained transparency without obvious edema or immune rejection and displayed a normal corneal thickness. CENCs formed a continuous, functional monolayer exhibiting native marker expression. However, significant edema, fibrosis and loss of transparency were evident in grafts lacking CENCs, emphasizing the essential contribution of cells to graft efficacy in endothelial repair.

Future perspectives: The only two studies addressing restoration of corneal endothelial function that met the inclusion and exclusion criteria for this review reported a comprehensive evaluation of the transplanted cornea. Nevertheless, extended follow-up periods are warranted in future studies assessing other biomaterial-based approaches.

### 3.5. Assessment of Risk of Bias

**Figure 2:**
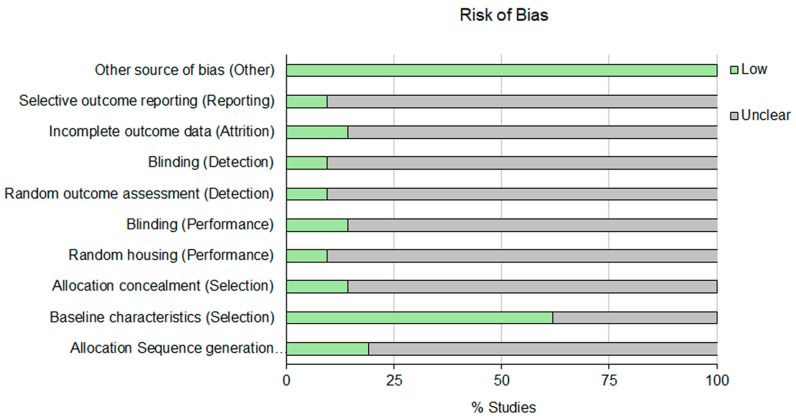
Risk of bias assessment of the included studies.

Among all the evaluated sources of bias, selection bias, specifically related to the reporting of animal characteristics, was considered to pose the lowest risk. Thus, 62% of the studies clearly did specify the animal species, sex, gender and weight of the subjects that underwent keratoplasty to evaluate the efficacy and safety of corneal implants. In contrast, sufficient information concerning the remaining subjects analyzed was only provided in 20% of the studies (Figure 2). Reviewers did not find any other issue susceptible to being considered as risk of bias.

## 4. Discussion

The scarce availability of donor corneas presents a significant obstacle in the clinical management of corneal injuries requiring transplantation. Bioengineered corneal substitutes offer a promising solution to this unmet medical need. This systematic review analyzes the progress in artificial cornea engineering over the last ten years. Most engineered constructs have been developed for use in corneal stromal and/or epithelial layer restoration, whereas only a limited number of grafts, based on just three and two biomaterials, have been explored for epithelial and endothelial regeneration, respectively.

Overall, the studies demonstrated favorable outcomes in terms of biocompatibility and efficacy in the regeneration of corneal epithelial, stromal and endothelial layers, with artificial corneal stromal substitutes, both with and without epithelium component, being extensively evaluated, whereas research in the field of corneal epithelial and endothelial grafts is still scarce.

**Figure 3:**
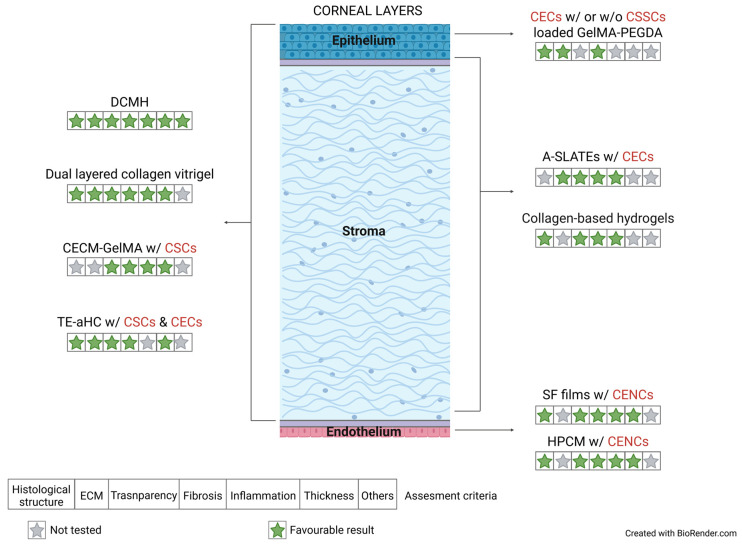
Schematic illustrating the most promising corneal grafts for the regeneration of each corneal tissue layer. A-SLATEs: Aligned self-lifting analogous tissue equivalents; CECs: corneal epithelial cells; CECM: corneal decellularized extracellular matrix from fresh porcine corneas; CENCs: corneal endothelial cells; CSCs: corneal stromal cells; CSSCs: corneal stromal stem cells; DCMH: decellularized corneal matrix hydrogel; GelMA: gelatin methacrylate; HPCM: human purified type I collagen membrane; PEGDA: poly (ethylene glycol) diacrylate; SF: Silk Fibroin; TE-aHC: tissue-engineered human anterior hemi-cornea.

Generally, the success of biomaterials employed for corneal epithelial regeneration remains insufficiently characterized, given that assessments of key parameters such as corneal transparency or thickness have not been performed and results relied exclusively on histological analysis in two of the three analyzed studies. Moreover, the duration of post-transplant follow-up was lower than one month, which may affect the robustness of the observed outcomes. Therefore, although a more in-depth evaluation is still pending, the inclusion of CEC or both CEC and CSSC into a GelMA-PEGDA matrix appears to be a promising strategy for corneal epithelial regeneration, as evidenced restored epithelial structure without signs of fibrotic response (Figure 3). This option might be especially valuable in the treatment of the Limbal Stem Cell Deficiency (LSCD), particularly, in cases of significant corneal scarring and LSCD involving the visual axis, where there is often need for a keratoplasty for visual rehabilitation. Moreover, complications of chemical and thermal injuries of corneal epithelial layer causing scarring and opacification of the cornea could be surgically managed using these types of grafts in the keratoplasty.

A wide range of scaffold biomaterials derived from extracellular matrix components has been extensively evaluated for the regeneration of both the corneal stromal and epithelial layers, while collagen- and fibrin-based materials have also been investigated for this purpose. A cellular component was present in 60% of the artificial grafts. Considering the regenerative outcomes of all the bioengineered corneas analyzed, aPCS, both acellular and recellularized DPC constructs and 3D-BDCS scaffolds proved to be unsuccessful approaches, as they resulted in unacceptable levels of corneal opacity, abnormal thickness and adverse fibrotic or immune response. In contrast, the DCMH, CECM-GelMA and TE-aHC extracellular matrix-derived grafts and the dual layered collagen vitrigel scaffold, rank among the most promising engineered corneal substitutes, as summarized in Figure 3. Only two of them include cells as part of their composition (CECM-GelMA and TE-aHC) (Figure 3). Notably, the recellularized porcine corneal stroma scaffold (TE-aHC) was evaluated over the longest follow-up period (12 months). Using these types of constructs in the therapy of anterior lamellar corneal disorders, where both the corneal epithelium and stroma were damaged by deep trauma, burns, or infections, the subsequent scarring could be prevented by regenerating a transparent stroma.

After a corneal insult, quiescent keratocytes in the stroma were activated and trans-differentiated into metabolically active opaque light-scattering corneal myofibroblasts (CMFs). Upon completion of corneal healing, CMFs must disappear from the stroma. However, severe corneal trauma/injury often leads to undue generation and persistence of CMFs resulting in the loss of corneal transparency. In such cases, corneal transplantation utilizing artificial grafts may represent the most advantageous option. Biomaterials of diverse origin such as decellularized extracellular matrix, collagen derivatives, reduced graphene oxide-reinforced titania-based composites and scaffold-free cell sheets have been explored with corneal stromal regenerative purposes. Corneal cells were included in three of the ten scaffolds evaluated. Overall, no inflammatory events following keratoplasty as well as transparency preservation during the follow-up time were reported for any of the evaluated scaffolds. Similarly, no observable structural and functional differences with respect to normal corneas were observed when histological evaluation was performed. However, the fibrotic response after transplantation did not be assessed for half of the biomaterials tested. In summary, A-SLATES and all four analyzed composite collagen-based hydrogels (MBPC thin, MBPC thick, HBPC and MHBPC thick) may be considered as the most promising approaches for corneal stromal tissue substitution, since both biomaterials did not promote fibrosis or inflammatory responses post-surgery, while preserving transparency and a histological structure resembling that of the healthy native cornea (Figure 3). Numerous corneal diseases, including dystrophies and ectatic disorders such as keratoconus, which compromise the physiological anatomy and transparency of the corneal stroma, may also benefit from corneal transplantation procedures employing this type of graft.

Regarding the innermost corneal layer, endothelial dysfunction, secondary to aging, intraocular surgery, infections or graft rejection, trauma and hereditary corneal diseases, continues to be a primary indication for corneal transplantation (42%). Endothelial cell incorporation to silk fibroin and human purified type I collagen have been evaluated for corneal endothelium replacement. A comprehensive assessment focused on the impact of endothelial cell inclusion into scaffolds was performed. In both cases, the endothelial cell incorporation resulted in the complete restoration of corneal transparency following a postoperative interval and a similar corneal thickness when compared to its healthy contralateral cornea. Artificial cellularized grafts did not elicit inflammatory or fibrotic responses and exhibited cell density and functionality comparable to those of healthy control eyes, as evidenced by the expression of characteristic endothelial markers (Figure 3). In contrast, the regenerative process was completely hindered when acellular scaffolds were transplanted, reinforcing the importance of cell-seeded constructs in corneal endothelial regenerative strategies.

Overall, decellularized corneal tissues derivatives have emerged as promising graft due to their contribution to nature recognition signals as well as their mechanical properties [59]. In contrast, batch to batch variability and the use of detergents and nucleases in the decellularization process may provide a barrier to the clinical application of this biomaterial [60]. On the other hand, scaffold features such as transparency, controllable biodegradability and biocompatibility provided by collagen derivatives position it as a potentially favorable alternative to be used in corneal surgery with regenerative purposes [61,62]. However, the mechanical weakness and limited elasticity of collagen remain key challenges that need to be overcome in the fabrication of effective scaffolds [63]. In addition, in order to better mimic the anatomical and structural characteristics of the cornea, integration of emerging technologies such as 3D bioprinting into the manufacturing process of corneal implants should be considered [20,64,65,66]. To date, only a limited number of in vivo studies assess the efficacy of corneal grafts fabricated by this promising technology. Other concerns, such as the use of surgical sutures and the choice of the keratoplasty technique are additional variables that may significantly impact the outcome of the regenerative process [67,68]. Finally, most regenerative corneal substitutes that demonstrate enhanced clinical outcomes incorporate corneal cells. However, further research is needed to evaluate the exact impact of incorporating distinct corneal cell phenotypes into scaffolds, given the limited number of in vivo studies comparing the regenerative efficacy of the acellular versus cell-loaded versions of the same biomaterial [39,42,48,51]. Moreover, the use of extracellular vesicles (EV) and plasma rich in growth factors in these engineering strategies need to be explored [69,70,71]. In this context, crosstalk between the epithelium and stroma mediated by EVs has already been described [72]. In particular, EV enriched in microRNAs (miRNAs), short non-coding RNAs that modulate gene expression through translational repression or mRNA degradation, have emerged as potent regulators of critical biological processes, including wound healing, nerve regeneration and the modulation of inflammatory responses. Accordingly, EV-associated miRNAs may represent a promising and potentially transformative approach for the development of personalized corneal therapeutic strategies [73].

Results of the risk of bias assessment highlight the need for enhanced methodological transparency and reporting standards in in vivo studies focused on bioengineered grafts for corneal regeneration. No information on the assessed risk-of-bias items, other than baseline characteristics of the animals included in the in vivo studies, was available for more than 75% of the studies reviewed. This insufficient methodological and reporting transparency substantially compromises the robustness of the conclusions that may be drawn from the analysis of the reported outcomes. Accordingly, the regenerative potential of the corneal grafts should be interpreted with caution. Notably, no risk of bias was identified in 70% and 60% of the evaluated parameters in studies investigating DCMH and CECH-GelMA scaffolds, respectively, for the regeneration of both epithelial and stromal corneal layers.

## 5. Conclusions

A rigorously reported comparison between engineered corneal tissue alternatives and healthy, native corneas, focusing on optical properties (transparency and light scattering), tissue quality (inflammation and fibrosis) and anatomical fidelity, is essential to advancing the development of clinically viable corneal substitutes. Collectively, these findings contribute to the growing body of evidence supporting the potential of tissue-engineered corneal substitutes as viable therapeutic options for corneal blindness and vision impairment.

## Figures and Tables

**Table 1:** Summary information of the references included in this review.

Corneal Layer to Be Regenerated	Reference	Graft	Bioprinting	Scaffold Matrix Composition	Cell Inclusion	Toxicologic/Biocompatibility Assays	Cell Viability/Functionality In Vitro Assays	Scaffold Suturability
EPITHELIUM	Wang L et al., 2025 [39]	GelMA-PEGDA	✓	Gelatin methacrylate and poly (ethylene glycol) diacrylate	hCECs and hCSSCs	✗	Live/dead staining Ki67, CK3 y LUM	✓
	Hong S et al., 2018 [46]	PLGA	✗	Poly(lactic-co-glycolic) acid membranes	hCjECs and hTFs	✗	Live/dead assay at 7 and 14 days. Cell proliferation and gene expression were also determined.	✓
	Hyun DW et al., 2014 [50]	Biomaterial-free cell sheet	✗	✗	Human oral mucosal epithelial cells and limbal epithelial cells	✗	Viability and proliferative capacity assay	✗
EPITHELIUM and STROMA	Nie X et al., 2024 [54]	rhEGF/TSA-loaded bilayer graft	✓	Concentrated aqueous solutions of Gel/Alg-CDH.	✗	✗	NA	✓
	Chameettachal S et al., 2023 [40]	DCMH	✗	Human and bovine decellularized corneas	✗	✓	NA	✗
	Zhang M et al., 2023 [41]	CECM-GelMA	✓	Gelatin methacrylate and corneal decellularized extracellular matrix from fresh porcine corneas	hCFs	✓	Live/dead assay after culturing for 1, 7 and 14 days	✗
	Blanco-Elices C et al., 2023 [56]	OAC HAC	✗	Nanostructured fibrin-agarose biomaterial	Primary cell cultures of corneal stromal cells (immersed) Primary cell cultures of human corneal epithelial cells (surface) andHWJSC (surface)	✓	✗	✓
	Fernández-Pérez J et al., 2021 [42]	DPC	✗	Decellularized porcine corneas	Isolated human stromal keratocytes (fibroblasts)	✗	Stromal markers detection: ALDH3A1, CD34, KERA, decorin, LUM and Col-I	✓
	Wang XK et al., 2020 [57]	Dual-layered collagen vitrigel	✗	sBM and a sSL containing tissue-derived ECM microparticles	✗	✗	NA	✓
	Rico-Sánchez L et al., 2019 [43]	TEAHC	✗	Fibrin–agarose nanostructured sheets	Primary cell cultures of epithelial cells and stromal keratocytes	✓	EPXMA to determine cell viability. mRNA expression of the cell proliferation marker PCNA, the pro-apoptotic gene CASP9 and crystallin gene LDHA, involved in corneal transparency	✓
	Park J et al., 2019 [44]	3D-BDCS	✓	Decellularized corneal ECM	hTMSCs differentiated to keratocytes	✓	NM	✓
	Liu J et al., 2019 [55]	DLPCS	✗	Decellularized lamellar porcine corneal stroma	✗	✗	NA	✓
	Xu B et al., 2017 [58]	TE-aHC	✗	aPCS	ntHCS and ntHCEP	✗	Immunofluorescent staining for CK3, vimentin, ALDH3A1, E-cadherin, connexin 43, integrin b1, Na1/K1-ATPase a1 polypeptide, and Ca21-ATPase	✓
STROMA	Li Z et al., 2019 [45]	1rGO/TiO_2_ 1rLCGO/TiO_2_	✗	Reduced graphene oxide-reinforced titania-based composites	✗	✓	NM	✓
	Syed-Picard FN et al., 2018 [47]	Cell tissue sheet	✗	✗	hCSSC	✗	Structural evaluation, immunostaining for ECM: Col-I, Col-V and KERA	✗
	Gouveia RM et al., 2017 [49]	Self-Lifting Analogous Tissue Equivalents: Aligned: A-SLATEs Randomly oriented: R-SLATEs	✗	Corneal stromal tissues formed by cells and deposited ECM	Human corneal epithelial (for seeding onto the scaffold) and stromal cells (for obtaining the scaffold)	✗	Live/Dead Cell double staining kit	✓
	Hashimoto Y et al., 2016 [52]	DPC-HHP	✗	Porcine cornea decellularized by HHP method.	✗	✗	NA	✓
	Rafat M et al., 2016 [53]	MBPC thin MBPC thick HBPC MHBPC thick	✗	Composite collagen-based hydrogels with a centrally transparent core and embedded peripheral skirt of adjustable transparency and degradability	✗	✓ (in vitro)	NA	✗
ENDOTHELIUM	Vázquez N et al., 2017 [48]	SF films	✗	SF-based artificial graft	rCENCs	✗	SEM (polygonal morphology) Staining for ZO-1 and Naþ/Kþ ATPase	✓
	Vázquez N et al., 2016 [51]	HPCM	✗	Human purified type I collagen membrane, isolated from remnant cancellous bone chips	Rabbit corneal endothelial cells	✗	✗	✓

Abbreviations: ALDH3A1: aldehyde dehydrogenase 3 family member a1 polypeptide; aPCS: cellular porcine corneal stromata; CASP9: caspase-9; CDH: carbohydrazide-modified alginate; CECM: corneal decellularized extracellular matrix from fresh porcine corneas; CK3: cytokeratin-3; Col-I: collagen type I; Col-V: collagen type V; DCMH: decellularized corneal matrix hydrogel; DPC: decellularized porcine corneas; ECM: extracellular matrix; EPXMA: electron-probe X-ray microanalysis; Gel/Alg-CDH: gelatin and carbohydrazide-modified alginate; GelMA: gelatin methacrylate; HAC: heterotypical bioartificial corneas; hCECs: human corneal epithelial cells; hCFs: isolated human corneal fibroblasts; hCjECs: human conjunctival epithelial cells; hCSSCs: human corneal stromal stem cells; HHP: high hydrostatic pressure; hTFs: human Tenon’s fibroblasts; hTMSCs: human turbinate-derived mesenchymal stem cells; HWJSC: Umbilical cord Wharton’s jelly stem cells; KERA: keratocan; LDHA: lactate deshidrogenase A; LUM: lumican; NA: not applicable; Naþ/Kþ ATPase: sodium-potassium ATPase; NM: not mentioned; ntHCEP: nontransfected human corneal epithelial cells; ntHCS: nontransfected human corneal stromal cells; OAC: orthotypical bioartificial corneas; PEGDA: poly (ethylene glycol) diacrylate; PCNA: Proliferating Cell Nuclear Antigen; PLGA: poly(lactic-co-glycolic) acid; rCENCs: rabbit corneal endothelial cells; rhEGF: recombinant human epidermal growth factor; sBM: Biomimetic synthetic Bowman’s membrane; SEM: scanning electron microscopy; SF: silk fibroin; SLATEs: self-lifting analogous tissue equivalents; sSL: a synthetic stromal layer; TE-aHC: tissue-engineered human anterior hemi-cornea; TEAHC: tissue-engineered anterior human cornea; TSA: Trichostatin A; ZO-1: Zonula Occludens-1; ✓ If “yes”; ✗ If “not”.

**Table 2:** Summary of the characteristics of the studies and obtained results of graft transplantation for corneal epithelial layer regeneration.

Graft	Graft Options	Outcomes Summary						
		Inflammation	Fibrosis	Neovascularization	Transparency	Thickness	Histological Examination	
							Structural Features	ECM Components
GelMA-PEGDA [39]	CEC/CSSC-loaded (A) CEC-loaded (B) cell-free (C)	NT	✗ (A, B) ✓ (C)	✗ (A, B) ✓ (C)	NT	NT	Epithelial cells arranged regularly (A, B) No goblet cells (conjunctive) (A, B) Goblet cells (C)	NT
PLGA [46]	PLGA + epithelial tissue sheet (A) PLGA (B)	NT	NT	NT	NT	Partially degraded (B)	Re-epithelialization (A)	NT
Biomaterial-free cell sheet [50]	with fibrin support (A) without fibrin support (B)	NT	NT	NT	NT	NT	Positive expression of p63, ABCG2 and Ki-67 (B) multiple layers of epithelial cells without visible goblet (B)	K3, K4 and K13 were partially expressed in the transplanted epithelial cell layers (B).

**Abbreviations**: CECs: Human corneal epithelial cells; CSSCs Human corneal stromal stem cells; GelMA: Gelatin methacrylate; K3: Cytokeratin-3; K4: Cytokeratin-4; K13: Cytokeratin-13; PEGDA: poly (ethylene glycol) diacrylate; PLGA: Poly(lactic-co-glycolic) acid; NT: Not tested; ✗: negative results; ✓: positive results.

**Table 3:** Summary of the characteristics of the studies and obtained results of corneal epithelial and stromal layer regeneration after graft transplantation.

Graft	Graft Versions	Cellularized Option	Outcomes Summary						
			Inflammation	Fibrosis	Optical Properties	Transparency	Thickness	Histological Examination	
								Structural Features	ECM Components
DCMH [40]	NT	✗	✗	✗	Stromal reflectivity and mean radius of curvature measurement close to pre-operative values.	Recovered.	No significant differences.	Similar total number of epithelial cells. Densely packed and highly organized ECM fibrils in the entire cornea.	Stromal matrix synthesis and restoration of milieu. Col I detection and increased LUM expression.
CECM-GelMA [41]	NT	✓	✗	✗	NT	Recovered at 2 months	No significant differences.	NT	NT
DPC [42]	DPC (A) DPC + human stromal keratocytes (B)	✓ (B)	NT	✓ (A, B).	NT	Did not return (A, B).	NT	Complete epithelial covering (A, B). Cells sparsely distributed in the central scaffold (A, B). Increased density of cells in the anterior area (regeneration) (A, B).	Positive cell staining for ALDH1A1 in the epithelium, the native stroma and endothelium (A, B).
TEAHC [43]	NT	✓	Initial inflammatory process disappearing 3 to 6 months post-surgery.	NT	NT	Improvement of transparency levels.	NT	Graft properly integrated, displaying a proper structural integrity and cells inside keeping their adequate corneal differentiation status.	NT
3D-BDCS [44]	NT	✓	Corneal edema decreased over time.	NT	NT	NT	Overall thickness reduction.	NT	NT
rhEGF/TSA-loaded bilayer graft [54]	rhEGF/TSA-loaded bilayer graft (A) Hydrogel scaffold (B)	✗	NT	✗ (A). ✓ (B).	NT	Maintained a good optical transparency (A, B).	Epithelial layer thickness higher than (B) and comparable to (A) normal tissue.	An integrated epithelium-stroma structure (A, B). Re-epithelialization (A, B). Epithelial cells more tightly aligned (A). Appropriate stroma thickness (A, B).	Regenerated corneal stroma consisting of collagen fibers (A, B). Epithelial layers expressed CK3 protein (A, B). Parallel Col I fibers and LUM expression (A).
DLPCS [55]	NT	✗	Slight edema at 1-month post-operation gradually disappeared. Without activated cells notably infiltrating the stroma.	NT	NT	At 24 ± 5 days sufficient transparency.	Decreased gradually to a normal thickness.	Lower stromal and epithelial cell density.	NT
Nanostructured fibrin-agarose biomaterials with corneal stromal cells (immersed) and an epithelial-like layer on top [56]	OAC (A) HAC (B)	✓ (A, B)	NT	NT	NT	Opacity comparable to control native corneas (A). Opacity decreased with time (B).	NT	Appropriate stroma and epithelial layers (A, B). Well-differentiated epithelium with numerous cell strata (A, B). Dense corneal stroma consisting of numerous lamellae of well-organized fibers with abundant stromal cells (A, B).	Well differentiated stroma (increased contents of collagen, proteoglycans, decorin, KERA and LUM, higher levels of maturation and spatial organization) (A, B).
Dual-layered collagen vitrigel [57]	NT	✗	✗	✗	NT	Remained clear at 30 days post-surgery.	Complete restoration of corneal thickness.	Complete re-epithelialization.	Functional multilayer with limbal stem cell marker p63. Expression through the layers of K3 and K12 epithelial markers and tight junction protein ZO-1 K14 expressed in the lower layer.
TE-aHC (tissue-engineered human anterior hemi-cornea) [58]	TE-aHC (A) aPCS (B)	✓ (A) ✗ (B)	Corneal edema at 1-month post-surgery decreased (A). Intense corneal edema and neovascularization (B).	✗ (A, B)	NT	Almost identical to normal control eyes from day 60 to day 360 (A). Remained turbid (B).	No significant difference (A). Significantly thinner (B).	Native-like epithelium together with a regularly arranged stroma with sparsely distributed keratocytes (A). Few keratocytes but a neoregenerated epithelium (B). Appropriate cell morphology (A).	Appropriate amount and distribution pattern of GAGs and collagen fibrils (A, B).

Abbreviations: ALDH1A1: aldehyde dehydrogenase 3 family member α1 polypeptide; aPCS: acellular porcine corneal stromata; CECM: corneal decellularized extracellular matrix from fresh porcine corneas; Col I: Collagen type I; DCMH: decellularized corneal matrix hydrogels; DLPCS: decellularized lamellar porcine corneal stroma; DPC: decellularized porcine corneas; ECM: extracellular matrix; GAGs: glycosaminoglycans; GelMA: gelatin methacrylate; HAC: heterotypical bioartificial corneas; KERA: Keratocan; K3: Keratin 3; K12: Keratin 12; K14: Keratin 14; LUM: lumican; NT: Not tested; OAC: orthotypical bioartificial corneas; ZO-1: Zonula Occludens-1; ✗: negative results; ✓: positive results.

**Table 4:** Summary of the characteristics of the studies and obtained results for corneal stromal regeneration after graft transplantation.

Graft	Graft Versions	Cellularized Option	Outcomes Summary					
			Inflammation	Fibrosis	Optical Properties	Transparency	Histological Examination	
							Structural Features	ECM Components
Reduced graphene oxide-reinforced titania-based composite [45]	1rGO/TiO_2_ (A) 1rLCGO/TiO_2_ (B)	✗ (A, B)	✗	✗	NT	NT	Epithelium remained intact (A, B). Appropriate stromal cell density and morphology (A, B).	NT
Cell tissue sheet (CSSC from human limbal tissue and differentiated into keratocytes) [47]	NT	✓	NT	NT	Normal light scattering of corneal stromal tissue containing tissue sheets.	Transplanted corneas qualitatively appear transparent.	Transplanted corneas maintained a structure similar to normal corneas.	Human cells in mouse eyes were functional (producing keratocan).
SLATEs [49]	In terms of tissue organization: aligned, A-SLATEs (A) randomly oriented, R-SLATEs (B).	✓ (A, B)	✗	✗ (A) ✓ (B)	NT	Corneas remained clear and haze-free (A, B).	NT	Grafts showed appropriate vimentin-positive cells (A, B).
DPC-HHP [52]	NT	✗	✗	NT	NT	Slight disorganization of collagen fibrils did not affect corneal transparency.	Graft did not undergo remodeling.	NT
DPC-HHP [52]	NT	✗	✗	NT	NT	Transparency was maintained.	Graft did not undergo remodeling. Highly organized graft structure and similar to the host cornea.	NT
Composite collagen-based hydrogels [53]	MBPC thin (A) MBPC thick (B) HBPC (C) MHBPC thick (D)	✗ (A, B, C, D)	Initial graft population with CD45—bone marrow-derived cells such as macrophages (A, B, C, D).	Initial graft population with host cells (α-SMA—myofibroblasts).	NT	Postoperative haze in biomaterial-implanted corneas diminished over time (A, B, C, D).	Postoperatively coverage of implanted corneas by epithelium (A, B, C, D). The corneal epithelium derives innervation (A, B, C, D). No significant difference in sub-basal nerve density comparing to native cornea (A, B, C, D)	NT

**Abbreviations**: α-SMA: alpha-smooth muscle actin; CSSC: human corneal stromal stem cells; DPC: decellularized porcine corneas; HHP: high hydrostatic pressure; NT: not tested; SLATEs: self-lifting analogous tissue equivalents; ✗: negative results; ✓: positive results.

**Table 5:** Summary of the characteristics of the studies and obtained results for cornea endothelial layer regeneration after graft transplantation.

Graft	Graft Versions	Outcomes Summary					
		Inflammation	Fibrosis	Transparency	Thickness	Histological Examination	
						Structural Features	ECM Components
SF films [48]	SF films with rabbit CENCs (A) Acellular SF (B)	✗ (A) Corneal edema (B)	✗ (A) Slightly marked fibrotic tissue (B)	Transparency was restored (A). Transparency was not restored (B)	Normal corneal thickness (A). Enhancement in corneal thickness (B)	A monolayer of rabbit CENCs showed ZO-1 and Naþ/Kþ ATPase markers, (proper intercellular junctions and cellular pump function) (A). Normal endothelial cell count (A).	NT
HPCM, isolated from remnant cancellous bone chips [51]	HPCM with CENCs (A) Acellular HPCM (B)	✗ (A) Corneal edema (B)	✗ (A) Slightly marked fibrotic tissue (B)	Transparency at day 10 subsequently maintained (A). Corneal transparency was not restored (B).	Normal corneal thickness (A). Enhancement in corneal thickness (loss of corneal endothelial functionality) (B)	Graft attached tightly to the corneal stroma. A continuous monolayer of rabbit CENCs with normal morphology and phenotypical markers (ZO-1 and Na+/K+ ATPase) (A). Loss of corneal endothelial functionality (B).	NT

Abbreviations: CENCs: corneal endothelial cells; SF: silk fibroin; HPCM: human purified type I collagen membrane; ZO-1: Zonula occludens-1; Na+/K+ ATPase: sodium-potassium ATPase; NT: not tested; ✗: negative results.

## Data Availability

Dataset available on request from the authors.
